# Qualitative Investigation of Challenges for Students with Learning Disabilities Attending Saudi Universities

**DOI:** 10.3390/bs16040521

**Published:** 2026-03-31

**Authors:** Mohaned G. Abed, Todd K. Shackelford

**Affiliations:** 1 Department of Special Education, Faculty of Education, King Abdulaziz University, Jeddah 21589, Saudi Arabia; mabed@kau.edu.sa; 2 Department of Psychology, Oakland University, Rochester, MI 48306, USA

**Keywords:** learning disabilities, Saudi Arabia, qualitative research, higher education, university

## Abstract

Students with learning disabilities (LDs) are members of most educational settings, from primary school to university. At the university level, students with LDs face many challenges. The aim of the current study is to explore the challenges and needs of students with LDs attending Saudi Arabian universities. The study investigates factors contributing to the academic and social successes of students with LDs. Data were collected using a qualitative interview methodology. The sample comprised nine participants: five students diagnosed with LDs and four faculty members. The results suggest that students with LDs continue to face challenges and often do not receive adequate support from the social and academic environments within the university, and this can negatively impact their educational performance. These results contribute to a better understanding of the needs of students with LDs in higher education and may assist in the development of a more inclusive university environment, particularly in Saudi Arabia.

## 1. Introduction

A university’s primary goals are to enhance scientific research, deepen academic knowledge, and meet the educational needs of students (Riddell & Weedon, 2014). In modern society, higher education is a pathway to professional and personal advancement. It is for this reason that the availability of inclusive education in institutions of higher learning is an important objective (Ainscow, 2020; Aiello et al., 2019; Bartolo et al., 2025). Although opportunities for students with learning disabilities (LDs) to enroll in university have improved in the recent past, Newman et al. (2011) estimated that these opportunities are still just half of those of their counterparts without LDs. Indeed, a recent systematic review by Gull et al. (2025) identified 14 key thematic barriers, including negative attitudes, inadequate infrastructure, and limited support services, which continue to hinder the success of students with disabilities in higher education across diverse international contexts. The commitment to inclusive education is also reflected in the United Nations 2030 Agenda for Sustainable Development, which pledges not to leave anyone behind. Such a commitment includes students with LDs. These students have the right to benefit from opportunities provided by higher education, which should provide them with a platform to refine their skills, address challenges, and embrace distinct styles of learning. The university plays a role in facilitating the integration of students with LDs into professional and social networks, promoting diversity and inclusivity in educational and employment settings.

LDs reflect a heterogeneous set of disorders expressed as challenges in, for example, acquiring and using mathematical reasoning, writing, reading, speaking, and listening. The heterogeneous nature of LDs is acknowledged by the National Joint Committee on Learning Disabilities (1990), which adds that these disorders are inherent to the individual affected, are suspected to be linked to the central nervous system, and may occur across an individual’s lifespan. In Saudi Arabia, the Ministry of Education defines LDs as disturbances in one or more of the processes facilitating comprehending and using spoken or written language. Such disturbances may occur in mathematics, reading and writing, speaking, thinking, and listening, and are not a result of family care, learning conditions, mental or audio-visual disabilities, or other types of disabilities (Abed & Shackelford, 2020). Although it is widely accepted that intervention can assist students with LDs in developing strategies to learn more effectively, there is still a requirement for unique types of support across students’ learning journey, particularly as they move from secondary school to university education (Crawford, 2012). Scholars have validated the principle that providing suitable strategies and the required support to students with LDs facilitates their learning and increases their chances for academic success (Collinson & Penketh, 2010; Ingesson, 2007).

In educational contexts, LDs refer to neurological conditions that hinder the ability to process or store information. This definition is embraced by Simpson and Spencer (2021), who note the importance of distinguishing between LDs and learning problems. Learning problems refer to the effects of emotional disturbance, developmental disabilities, motor, visual, or hearing challenges, or to the effects of environmental, economic, or cultural disadvantages (Simpson & Spencer, 2021). Previous studies have documented no clear connection between LDs and motivation or intelligence (Sicherer, 2019). Studies also have documented that students with LDs sometimes outperform their peers without LDs (Lloyd et al., 2007). Previous research suggests that students with LDs can overcome challenges with reasoning, recalling, reading, spelling, writing, or organizing information, particularly when instruction follows traditional teacher-centered methods (Alquraini, 2011). Gibson (2012) identifies the most common LDs, including non-verbal LDs such as dysgraphia and dyscalculia.

Although the literature indicates that students with LDs are underrepresented in university education, their presence is increasing (Liasidou, 2014). This increase is associated primarily with students with “hidden” learning difficulties, including LDs, attention-deficit/hyperactivity disorder (ADHD), autism spectrum disorder (ASD), and dyslexia (Shea, 2019). Although LDs can affect the academic success of students before they enter university, the impact is more pronounced after entering university (Vogel & Adelman, 1992). Vogel and Adelman (1992) note that pre-university education often provides students with LDs with educational support that may not be as available for students attending university. Thus, support and programs for students with LDs should not be restricted to the earlier stages of education but should also be available at university (W. M. Hadley, 2007). Al-Korbi et al. (2024) found that around one-third of faculty members at a Middle Eastern university were unaware of the support services available to students with special educational needs, illustrating how gaps in institutional awareness can weaken inclusion efforts even when support policies have been established.

Although there has been an increase in awareness of LDs in recent years, there is a dearth of studies on the specific needs of students with LDs at the university level, particularly in the Middle East (Abed & Shackelford, 2020). The current study addresses this gap in the literature by exploring the challenges faced by students with LDs attending Saudi universities. The results may suggest recommendations for improving the support available to students with LDs, with special reference to the Saudi university context.

### Literature Review

Estimates indicate that there are about 1.5 million individuals diagnosed with a disability in Saudi Arabia (Saudi General Authority for Statistics, 2020). This estimate includes individuals with LDs. Other research suggests that the incidence of students with LDs is expected to increase across the world. For instance, Irwin et al. (2021) report that in the 2019–2020 school year, an estimated 33% of students in special education classes in the United States qualified as students with LDs.

A case study of university students with LDs by W. Hadley (2017) concluded that two main factors affect post-secondary success after students with LDs have been admitted to university. The first is that although access to higher education for students with LDs continues to increase, there is continued reluctance among students to disclose specific disabilities (W. Hadley, 2017). The second relates to the fact that LDs are often not obvious, in that there are no physical indicators of LDs. Student reluctance to disclose LD diagnoses is a concern, given that university students with LDs often must disclose such information if they are to receive support (Couzens et al., 2015).

For students with LDs to receive support, service providers at universities must establish strategies and goals for how they will support students to learn independently and make decisions relating to their educational experiences. Troiano (2003) acknowledges this view, contending that service providers should assist students in disclosing disability details when such information is needed. Rude et al. (2005) conducted research with the aim of exploring the perspectives of service providers who work with students with LDs. The same study also focused on the needs of students with LDs and the suitability of the educational services available to them. Greenfield et al. (2016) focused on the methods employed by teachers of students with LDs and their impact on student academic success. Keener and Bargerhuff (2006) investigated the experiences of teachers working with students with LDs at the university level, identifying the importance of teachers and students working collaboratively to positively impact students’ academic success.

A study conducted by Trainin and Andrzejczak (2006) aimed to identify mechanisms employed by students with LDs at universities to succeed academically. The results confirmed that students with LDs have specific needs and require support and specific programs to succeed in their academic activities. The same conclusion was reached by Alster (1997), who suggested that probationary periods of study may especially benefit students with LDs.

As a result of the increase in the number of students with LDs attending universities, the current study also explored Saudi university faculty members’ awareness of LDs, educational services available to students with LDs, the impact of their teaching methods on students with LDs, and their awareness of the educational environment as it may impact students with LDs. In addition, the current study explored the needs and services provided to university students with LDs, factors affecting their performance, and strategies to support them from the point of view of faculty members and the students themselves in Arab universities, in general, and in Saudi Arabia, in particular.

## 2. Methods

The present study employs a qualitative research design to explore the perspectives of students with LDs and of faculty regarding these students’ experiences at a university in Saudi Arabia. The specific method for gathering data is the semi-structured interview, and phenomenography was the selected approach. Phenomenography reflects a research strategy whereby methods are employed in examining phenomena (Marton, 1981). According to Marton (1981), the aim of phenomenography is to produce insights about experiences by identifying person-centered concepts in the immediate environment. Phenomenography places emphasis on the experienced, the conceptualized, and the apprehended (Marton, 1981). An important research outcome of phenomenography is to identify and organize expressed thoughts in the form of observations, opinions, and ideas (Punch & Oancea, 2014).

Punch and Oancea (2014) define phenomenography as a qualitative approach used to examine phenomena related to the way people understand a specific concept, with perception playing a significant role. The same authors add that phenomenography often includes detecting relationships between individuals and external factors in their environment. Using this approach, the present study focused on determining the needs of university students with LDs and the external factors affecting their educational experience. Use of this qualitative method allowed the researchers to explore strategies that might best support students with LDs attending Saudi universities, supporting the community service component of the research. Inspired by Marton (1981), the present study probes the notions, observations, and perceptions held by individuals, and then individually describes such notions and, where possible, organizes them into thematic categories. Following Marton, we suggest that there may be utility in considering both a phenomenological perspective and a thematic perspective. According to Marton, a phenomenological perspective affords the opportunity to capture individual-specific responses, whereas a thematic perspective affords the opportunity to consider whether these individual-specific responses might be organized into themes.

The research questions that guide the current investigation of the Saudi university context include: What are the needs of students with LDs at the university level? What factors influence the performance of students with LDs at the university level? What are the optimal strategies and actions for assisting students with LDs at the university level?

### 2.1. Research Approach

Semi-structured interviews were conducted to explore the perceptions of Saudi university students with LDs and of faculty in relation to the needs of these students for educational support. The sample included nine participants from King Abdulaziz University: five students with LDs and four faculty members. In addition to exploring the views of the participants about the needs of students with LDs, the study explored how the prevailing educational context impacts student performance. Participants were also asked to identify strategies for supporting students with LDs in achieving academic success.

### 2.2. Semi-Structured Interviews

The semi-structured interview is a tool for qualitative study that provides an informal and interpersonally welcoming method for data collection (Leavy, 2022). In the present study, the interview is conducted with the aim of gaining insight into the experiences of individuals, notably their unique perspectives. The method is valuable for its ability to capture a wealth of information related to the perceptions, views, attitudes, and experiences of individuals (Leavy, 2022). Thus, given that the aim of the current study was to describe the experiences and perceptions of Saudi university students with LDs and faculty that instruct these students, the researchers concluded that the qualitative semi-structured interview is an appropriate methodology. The development of the interview questions for this study was inspired by the arguments of Kvale and Brinkmann (2009), and additional details are provided in the sections below.

### 2.3. Sampling

Participants in the present study were identified using convenience sampling and through word of mouth. Available demographic details of the participants are presented in Table 1. The ages of participating students ranged from 20 to 25 years, and the ages of participating faculty members ranged from 35 to 55 years. The teaching experience of faculty members ranged between five and 20 years. No rewards were provided to participants. All participants completed a written statement of informed consent. Although participants were informed that participation was voluntary and that they could withdraw from the study at any time without the need to give a reason, no one withdrew. The Special Needs Center at King Abdulaziz University was targeted to recruit student participants. This targeting strategy ensured that participants were students attending the university and had documented LDs. Although the main aim of the study was not to produce results that could be generalized to a wider population, ensuring that the students who participated had documented LDs increased the validity and credibility of the study.

### 2.4. Procedure

Before recruiting participants, ethical approval was obtained from the Scientific Research Ethics Committee of King Abdulaziz University (Protocol 4527929, date of approval: 10 March 2023). Each participant provided written consent before data collection began. Throughout the study, the researcher ensured the privacy and confidentiality of each participant.

Pilot interviews were conducted with Saudi university students with LDs. Feedback from these pilot interviews informed modifications to the interview instrument to enhance clarity and understanding.

For the main study, potential interview participants were contacted by telephone and invited to participate. Upon consent, participants were encouraged to speak freely and confidently, as suggested by Kvale and Brinkmann (2009). Interviews lasted between 35 and 55 min and were recorded using a password-protected digital device, as recommended by Berg and Lune (2017). These recordings were transcribed verbatim and subsequently translated from Arabic to English by a local translator (see below for details).

According to Weinberg (2002), one key to successful interviews is for the interviewer to display empathy during the interview. To promote honest participation, the interviewer used a combination of summaries, empathic responses, and open-ended questions. This approach aims to motivate participants to share their authentic perspectives and to capture the phenomena under study more accurately. Kvale and Brinkmann (2009) also advocate the use of follow-up, elaboration, and probing questions, along with strategic silence, to enhance the validity of interviews. The primary goal of the interviews was to identify participants’ experiences and perceptions, and to learn about the relationship between these phenomena (Berg & Lune, 2017).

By adhering to ethical guidelines, developing and refining the interview instrument through pilot research, and using empathic and strategic interviewing techniques, this study aimed to collect accurate data on the experiences and perceptions of university students with LDs and their faculty in Saudi Arabia.

### 2.5. Data Analysis

Once the interview process was complete, the recordings were transcribed, and the ideas were organized into themes based on participants’ perceptions. The categorization of insights from the data was completed based on principles of phenomenography (Marton, 1981; and see Introduction, above). To identify perceptions and place them into thematic categories, the researchers re-read participant responses, as suggested by Weinberg (2002). By attending carefully to the transcribed text, the interviewer and senior author was able to better capture participant perceptions. This resulted in the interviewer identifying, interpreting, and labelling the thematic categories. Once the categorization was complete, the next stage was “meaning condensation,” which is conducted to abbreviate the meanings of what the participants reported, as suggested by Kvale and Brinkmann (2009). To present the key results (see below), the researchers pursued a balance in terms of providing interpretive commentary and presenting specific ideas shared by the participants verbatim, as suggested by Hennink et al. (2015).

#### 2.5.1. Analytical Process

Guided by phenomenographic principles (Marton, 1981; Whitfield et al., 2023), the analysis followed three iterative steps. First, data segments related to students’ experiences of learning with LDs were identified and condensed to form an initial pool of meaning. Second, these segments were compared and sorted to capture qualitative differences in how participants understood key issues. Third, the resulting patterns were organized into hierarchical categories that together formed an outcome space, where broader categories incorporated more specific variations (for example, institutional barriers that include individual-level challenges).

Variation was examined through repeated comparative readings, attending to dimensions such as separation (treating LDs as isolated from the wider context) and fusion (viewing support as integrated across systems), while keeping the number of categories parsimonious (around four to six) and logically related. This approach reflects phenomenography’s second-order focus on collective ways of experiencing a phenomenon, and differs from descriptive thematic analysis by emphasizing structural relationships among categories within the outcome space.

#### 2.5.2. Reflexivity

The interviewer and lead author—a Saudi academic with more than 10 years of experience in special education at King Abdulaziz University—brought insider knowledge of local higher education contexts, which helped build trust and rapport with participants. At the same time, the interviewer’s assumption that students with LDs are under-supported in universities created a risk of confirmation bias.

To address this, the interviewer engaged in regular peer debriefing with co-authors and maintained reflexive journals after each interview, noting how his position as a faculty member might shape the questions asked or the way he interpreted comments about institutional barriers. This reflexive stance deepened the contextual understanding of participant accounts while deliberately bracketing the interviewer’s preconceptions, so that participants’ perspectives and interpretations remained central to the analysis (Berger, 2015).

#### 2.5.3. Translation

All interviews were conducted in Arabic and were transcribed verbatim and then independently translated into English by a certified Arabic–English translator with experience in educational research. To ensure accuracy and equivalence of meaning, a second independent translator performed back-translation into Arabic, and discrepancies between versions were discussed collaboratively.

These discussions drew on a “validity horizon” perspective, directing attention to culturally loaded terms such as “support,” and seeking formulations that carried comparable meanings across languages rather than literal matches. The analysis was conducted using both the original Arabic transcripts and the English translations, treating translation as an interpretive process rather than a technical transfer of text, in line with recommendations for qualitative research in multilingual settings (Zhao et al., 2024).

## 3. Results

Based on the objectives and the questions guiding the present study (see above), a descriptive method was employed to present the results efficiently but accurately. The main aim of the method is to monitor a phenomenon as it is reported, which relies on gathering, categorizing, and analyzing data, and identifying relationships that help the researcher arrive at the meaning of the responses. We discuss the participant responses according to three main themes (see above).

### 3.1. Theme 1: Needs of Students with LDs

Participants 1–5 (all students) indicated that they needed training, guidance, advice, direction, motivation, and support that included technological, personal, and academic aspects. They added that they require a supportive study environment and effective communication with the university and its community. The students reported that they need accommodations and services that are appropriately prepared, including note-taking services, alternative testing sites, and extended examination times. Participants 1 and 2 indicated that they require customized academic support designed to meet their level of need. Included in that support are educational guidance and the provision of enhanced resources. Participant 4 commented that, “We need assistive technologies to make the study process more comfortable for us.” Participants 3 and 5 noted that they need to feel a sense of belonging in a study setting that supports them psychologically and morally. They indicated that academic advisors, teachers, and friends should be willing and able to provide moral and psychological support.

Among the faculty participants, Participant 7 commented that, “Students with learning disabilities may require training and personal support from professional counsellors or academic advisors to assist them in planning for courses or programs of study and develop organizational and study skills.” Participants 8 and 9 shared the view that awareness campaigns can play a significant role in improving the lives of students with LDs in terms of knowing how to support them, identifying their needs, and understanding their rights. Participant 6 reported that, “Students with learning disabilities require a chance to communicate and participate in student activities and the university community, which can assist them in building social relationships and developing communication and cooperation skills.” Participants 6–9 supported the view that students with LDs sometimes require experience in addressing challenges of daily activities. They added that students with LDs may need more time and attention, particularly since their performance is sometimes lower than their classmates in standard classrooms. These same participants commented that the unique needs of these students call for the intervention of special education teachers with appropriate skills and knowledge. They added that such teachers should be willing to employ innovative and creative methods of instruction.

### 3.2. Theme 2: Factors Impacting Performance

There was agreement among student and faculty participants that there are positive factors that support students with LDs and facilitate their studies. The students also noted negative factors that hinder their progress. For example, Participants 1 and 2 shared the view that challenges they face include understanding numbers, getting academic support, and involvement in activities during lessons. They noted that they sometimes found it challenging to adapt to the classroom and the strict teaching and assessment methods that some faculty use. They reported that they should be provided with an opportunity to review information at their own pace so that they can fully comprehend the content. Other difficulties noted by Participants 1 and 2 included challenges in taking notes, particularly when faculty spoke quickly or delivered a presentation at a quick pace. Added to this, they noted that some faculty were not prepared to adjust the requirements of their assignments or change their teaching methods to meet the needs of students with LDs.

Several participants reported that students with LDs sometimes are anxious to ask the teacher for help. During the interview, Participant 3 commented that, “Due to my lack of understanding during lectures, I am usually marginalized by my colleagues and members of the faculty. This often leaves me embarrassed and resorting to isolation. When I tried to solve my challenge by talking to my lecturers about my disability, I discovered that they did not know anything about it.”

Participants also commented on the impact of high school teachers. For example, Participant 9 said, “I believe that high schools have a crucial role in providing the necessary resources and skills to students with learning disabilities. This could ensure that they are well prepared for success in higher education.” This is a view acknowledged by Participants 6 and 7, who noted that high school teachers must play a significant role in ensuring that students with LDs are prepared to advance to the university level by providing them with guidance, support, and skills required for success in university studies. Participant 5 also commented about the role of the high school. In his own words: “In my case, my teachers at the secondary school had an important role in my motivation. I attribute my ability to get here today to them. However, we are far from perfect, as we need training and knowledge of the university environment.” Participants 2–4 agree with Participant 5’s views and added that although some faculty at the university were helpful in meeting their needs, this was not the case with all faculty. They attributed this situation to the fact that many faculty have limited knowledge of LDs. They suggested that the solution to this challenge is for teachers to be trained to find alternative methods to ensure that learning activities are diversified. Participants 4 and 5 shared this perspective, noting that there are teachers that support them. They commented that the benefits of this support include greater self-confidence and improved skills. They reported that because of this support, they are now able to complete assignments and are more prepared for examinations.

However, some students reported negative experiences. For example, Participant 3 said, “When I discovered that I was not coping, I was left with no choice but to be brave and approach my teacher to ask for help. However, from his reaction, I was left feeling stupid and afraid. This is because my teacher failed to realize that I need more time and information to understand tasks.” Participants 1, 2, 4, and 5 agreed with Participant 3, noting that they sometimes struggled to secure guidance and support from faculty. The student participants added that they face challenges in understanding academic subjects because of the methods used in the classroom and a lack of resources. All this, they noted, has a negative impact on their academic performance. The student participants also shared the perception that students with LDs sometimes face discrimination from faculty and students who do not understand their situation and the needs resulting from their LDs. They noted that this results in psychological pressure and increased stress.

This reality was also noted by several faculty participants. Participants 7 and 9 shared that students with LDs experience stress and anxiety because of social and academic pressure, which impacts their ability to concentrate, in addition to producing deficits in self-confidence. Participant 9 added, “Many students with learning disabilities face a lack of self-confidence in their own abilities, resulting in lower levels of determination and motivation. This situation is made worse by the fact that many of them are not even aware of the situation they face, in addition to being uninformed about their needs and how they should deal with them.” This view was corroborated by Participant 8, who said, “Usually, we have no idea that there are students with LDs in our classes because many students with LDs do not identify themselves as having LDs. This makes it a challenge to know which students need our support.”

### 3.3. Theme 3: Auxiliary Procedures

Interview participants, both students and faculty, shared the view that universities can and should implement systems that help students with LDs secure the support they need from their teachers. The student participants also indicated that, apart from being understood, they need their teachers to cooperate with them in accessing lectures and completing tests and in ensuring that the evaluation methods are appropriate for students with their type of LD. In relation to making the necessary support available, Participant 1 commented that, “After I got support from my teacher who provided feedback and motivation in both personal and academic matters, my performance improved a lot. I felt that the teacher really cared about me and what was happening in my life.” In the same vein, Participant 2 said, “My professor also provided me with support, which assisted me with my papers, and he told me that I have improved a lot compared to the last semester. That made me feel really good.”

Participants also indicated that they viewed their friends and classmates as part of their support system. For example, Participant 3 stated that, “A number of my friends have been a source of support and assistance when needed. However, there are some peers who have not been able to show understanding of our special situation, and we feel embarrassed or afraid to communicate or deal with them.” In this regard, Participants 1 and 2 shared the view that the relationships they have formed with people in their classes have created a source of mutual benefit. Notwithstanding, some participants (1, 2, 4) reported that the university and faculty should better appreciate the needs of students with LDs and make available assistance and support for them to succeed academically. They added that universities need to provide diverse educational methods that can meet the needs of students with LDs. Open communication with different role players, including faculty, academic advisors, and peers, is another element the student participants emphasized, noting that this would assist them in securing support when they need it.

It is the view of Participant 9 that, “Students with LDs can be supported through the psychological and moral support of faculty members by encouraging them to effectively interact, communicate, and participate in activities with other students, in the process assisting them to improve their learning and self-confidence, and providing encouragement and appreciation.” Participant 8 noted that the support provided by role players “can assist students with LDs to develop their skills to participate in the university community as well as developing their study skills including using assistive technologies, searching for additional resources, using effective methods, time management, and being generally organized.” Participants 5 and 6 agreed with this view and added that making the required support available results from the effort and participation of peers, the academic community, faculty, and the university at large. These two participants noted that this support can help students with LDs achieve academic success and realize their potential.

Taken together, the three themes delineate an outcome space that captures qualitatively diverse ways in which students and faculty experience the needs of students with LDs and the conditions for their academic success. At one end of this outcome space, needs are framed as individual shortcomings that require greater personal effort, counselling, or remedial academic support. At the other end of this outcome space, they are understood as relational and institutional, involving psychologically safe learning environments, flexible teaching and assessment, and structured access to assistive technologies. Between these positions, participants also described contingent situations in which support depends on individual teachers’ goodwill or informal peer assistance rather than on institutionalized procedures, illustrating a spectrum that runs from medicalized conceptions of LDs to more socially and structurally oriented understandings. This pattern is visible in students’ accounts of feeling “stupid” or isolated when support is lacking, contrasted with reports of improved confidence and performance when faculty, advisors, and peers coordinate to provide appropriate accommodations and encouragement.

## 4. Discussion

In Saudi Arabia, the Ministry of Education is responsible for managing all stages of education, from primary school to the university level. Nonetheless, establishing services for students with LDs in Saudi Arabia has been slow. Alquraini (2011) contends that the release of the Regulations of Special Education Programs and Institutes (RSEPI) in 2001 was a turning point for students with LDs in Saudi Arabia. The same scholar notes that before then, there was a scarcity of LD programs. Although the university sector in Saudi Arabia represents great transformative potential, students with LDs continue to face barriers in the university environment. As was noted by the participants in the present study, these barriers range from a lack of effective communication, understanding, and awareness to deficits in experience and capabilities. The participants also revealed that faculty and other role players in universities have limited awareness of the needs of students with LDs, leading to misconceptions and inadequate support. This means that for universities to better serve students with LDs, they must acknowledge that such students need support to achieve personal and academic success.

The findings suggest that gaps in faculty awareness and institutional support for students with LDs remain a pressing issue in Saudi universities (Alsolami, 2024; Alrusaiyes, 2024). Participants described how cultural misconceptions and limited access to assistive technologies make the transition to university challenging for students with LDs (Alrusaiyes, 2024). They also pointed to Saudi-specific barriers, such as underused disclosure mechanisms despite increasing LD enrollments, and a mismatch between national policy commitments and the services students receive (Poch et al., 2023; Alrusaiyes, 2024).

These findings are consistent with international research showing that educational systems struggle with inadequate faculty training, yet they also suggest that the implementation of frameworks such as response-to-intervention has progressed more slowly in Saudi Arabia than in Western contexts (Poch et al., 2023; Alsolami, 2024). The results therefore challenge overly optimistic global narratives about inclusion by illustrating how, in the Saudi context, faculty attitudes—although improving—still limit equitable support because of ongoing resource and capacity constraints (Alrusaiyes, 2024).

Given this evidence of limited awareness and insufficient training, the study supports introducing mandatory, continuous professional development for university faculty about LD provisions and Universal Design for Learning principles (Alrusaiyes, 2024; Alsolami, 2024). Universities are positioned as responsible for LD screening, structured peer-support initiatives, and systematic rollout of assistive technologies, in line with both the 2000 Disability Welfare Law and the 2023 Rights of Persons with Disabilities Law, as well as recent Ministry directives (Poch et al., 2023; Alsolami, 2024). Policymakers are urged to embed clear accountability mechanisms so that these national strategies translate into consistent, effective practices on campus rather than remaining aspirational goals (Alsolami, 2024).

From the insights obtained from the results of the current study, it can be noted that, notwithstanding the reality that Saudi Arabian universities accept students with LDs in their programs, it is important for such institutions to make available to such students the necessary support at the behavioral, social, and academic levels. However, this should not be perceived as the exclusive role of universities but should also include efforts of policymakers at the level of government. The views of participants in the present study signal to policymakers to develop and implement policies that lead to practices that require institutions of higher education to identify students with LDs and ensure that they receive appropriate information and services, in keeping with the directives in the Disability Law in the Kingdom of Saudi Arabia for the year 2000.

In the present study, the participating students report that they sometimes experience a deficit of academic support, particularly regarding cognitive training, a type of support that is specialized, especially during the time of transitioning from secondary to higher education (W. Hadley & Satterfield, 2013). Students also noted the lack of training in study skills, an important aspect of assisting students with LDs manage amid the challenges inherent in higher education to improve their learning (Fuller et al., 2004). Additionally, the participating students report a shortage of assistive technology tools, which would help them better comprehend the material and constitute an important type of support to improve learning within the university environment (Crawford, 2012).

Among the barriers noted by the students in the present study are negative attitudes of students without LDs towards those with LDs, coupled with prejudicial attitudes of some faculty members (Madriaga, 2007). The views of students in the present study are in keeping with conclusions reached by Redpath et al. (2013), who noted that one challenge for students with LDs is that faculty sometimes lack awareness of the diverse needs of students. Students often must request adjustments to secure the required support, and even then such support may not be made available (Mortimore & Crozier, 2006). Such practices create obstacles to inclusion, usually disadvantageous to students with LDs when compared to their non-disabled counterparts.

Students in the current study noted that they understand that it may be challenging for their institutions and faculty to fully meet their needs because these needs are unique, and there are many students with varied LDs. Indeed, the population of students with LDs is growing, and Saudi Arabian faculty should receive more training, resources, and opportunities to be empowered to effectively educate students with LDs. Keller et al. (2016) acknowledge this view and add that this includes opportunities to acquire knowledge about the needs of students with LDs in teacher preparation programs.

As was pointed out by the participants, in instances in which there is inadequate support, cooperation, or understanding for students with LDs, coupled with discrimination, students may feel embarrassed about their condition and develop low self-confidence. The participants spoke about how these situations negatively affect their academic performance, the degree to which they integrate socially, and their psychological health. This can make them choose to isolate themselves. The data collected in the present study suggest that positive and effective support, collaboration, and communication with faculty and peers are essential elements in addressing challenges for students with LDs. This means that inclusive education should not just be made available because it is expected by the statutes but also because it is good for all students, as inclusive education can facilitate confidence, self-esteem, and social development, which can help in overcoming LDs and improve academic performance.

Also, it can be inferred from previous literature and the perspectives of participating students that awareness among faculty of LD issues is important. Kendall (2018) acknowledged this point and added that training teachers about disability issues should be mandatory and ongoing. It can also be concluded that the barriers faced by students with LDs are often external to their condition and focused on the learning environment (McManus et al., 2017). This point was supported by Hopkins (2011), who recommended disability awareness training for all teachers.

Participants also acknowledged academic barriers faced by students with LDs. These barriers include the attitudes of teachers, their reluctance to embrace alternative strategies of teaching, and difficulty accessing materials. Morgan (2023) acknowledged these impediments and added that they must be addressed to serve the goal of providing high-quality inclusive education to students with LDs. In relation to academic barriers, the previous literature and participants in the current study indicate that adapting content, processes, and methods continues to be challenging for faculty (Bunbury, 2020). This is a key point if one considers that both the literature and the participants agree that ensuring that resources and activities are adapted to the needs of the students with LDs is a key component of facilitating inclusive settings. More effective means of instruction include encouraging students to investigate, providing materials and notes in advance that will be used in class, and using reflective notes and PowerPoint presentations. Collins et al. (2019) add that providing materials ahead of the lesson may reduce the need for adjustments as the teaching activity develops. However, the same authors noted that this will not eliminate the need for reasonable adjustments. They noted that reasonable adjustments are based on the need to consider student diversity, in the process promoting the principle of equal opportunities and ensuring that the university does not discriminate against students who are different from the majority (Collins et al., 2019). Therefore, it can be concluded that the attitudes of faculty regarding students with LDs can either facilitate or hinder the students’ social and academic progress.

Together with the adaptation of teaching materials to ensure the inclusion of students with LDs, participants in the current study commented that inclusive methods can be employed by teachers in universities to replace or supplement traditional education methods. Encuentra and Gregori (2021) acknowledge this point and add that active and inclusive methods can improve learning by more effectively motivating students to participate and thus better understand concepts. However, the same authors note that, for this strategy to succeed, it is important to train university faculty on how to implement these strategies in the classroom. If implemented properly, this is a desirable option because it facilitates commitment and participation of students and faculty to develop a more welcoming attitude towards disability, which may encourage faculty to jettison traditional methods when these are less effective.

The results of previous studies, as discussed in the literature review, are consistent with those of the current study. Both the previous literature and responses of participants in the present study suggest that it is important to meet the immediate needs of students with LDs, together with providing access to support programs. For example, the results of the current study agree with those of Rude et al. (2005) and Keener and Bargerhuff (2006), which highlighted the need to provide programs for students with LDs so that they can overcome challenges. Both studies conclude that there is a need to teach more skills and provide training to deal with the challenges they meet.

Regarding improving the setting for students with LDs, scholars including Terenzini and Reason (2014) have used social-psychological models presented by Astin (1970, 1975, 1993) to develop more comprehensive models that can positively influence the development and educational outcomes of students. For example, the models involve the interaction between the characteristics and experiences of students before enrolling in universities—including social and individual experiences, academic preparation, policies, and faculty culture—and the peer environment in which the experience of the individual occurs, within and outside of the classroom. Acknowledging that successful outcomes rely not just on the student but also on the interaction between the student and the academic and social contexts of the university is in keeping with the conceptualization of LDs as a social phenomenon as opposed to exclusively an insufficiency inherent in the individual (Reid & Weatherly Valle, 2004).

The participating students noted that when they were in high school, positive relationships they had with their teachers and their preparation for the post-secondary phase through receiving guidance and advice, coupled with effective communication, had a strong positive impact on their adjustment to university. From this point of view, being aware of their disability, their own weaknesses and strengths, and the resources they need to succeed should serve as foci in discussions around individual support programs for students with LDs. Awareness of challenges faced by students with LDs can ensure that schools are clear about the assistance required by students with LDs before they transition to university (Townsend, 2007). The provision of training and support is a crucial element in preparing students with LDs for life at university. For instance, high schools can make available support related to skills for organizing their studies, conduct transition readiness assessments, help students develop post-secondary goals, provide a supportive and inclusive learning environment, and motivate students to work independently. Added to this, high schools can play a role in promoting awareness of issues related to LDs and encouraging effective communication, allowing students to interact with counsellors to secure the support they need. Such collaboration between students and high school teachers can help to ensure that students with LDs are prepared for success at university.

A study by Carter (2004) in the United States concluded that although participants were able to describe their LDs, not all of them could suggest ways of overcoming the obstacles they face to access the services and modifications required to achieve individual goals. This implies a need for students with LDs to receive training in self-advocacy. Other areas that could be suggested for training for these students based on the insights provided in the interviews include developing the attitudes, skills, and knowledge required for self-determination. The role played by peers and their cooperation in assisting students with LDs to succeed academically is highlighted in the present study. Both the literature and the results of the current study suggest elements related to the role of peers, including supporting students with LDs to secure professional attention, pursue effective collaboration with teachers, and engage in cooperative learning with other students.

To address obstacles faced by students with LDs in Saudi universities, participants in the current study suggested it is necessary to acknowledge and respond effectively to their distinct needs so that they can advocate for their needs and rights, access opportunities for peer support, communicate effectively, acquire social and academic skills, and boost self-confidence. This view is acknowledged by Jacklin (2011) and Richardson (2009), who comment that universities should make it easier for students to disclose their disabilities earlier so that support can be implemented sooner. Knott and Taylor (2014) concluded that there is a challenge in relation to this because students are often reluctant to disclose their disabilities. To deal with this challenge, Mortimore (2013) suggests that a culture change is needed, and academic institutions must act proactively to encourage students to disclose disabilities. The author adds that this could begin during university open days and career fairs.

Interpreted through inclusive education and disability studies perspectives, the findings suggest that many challenges reported by students with LDs in this Saudi university setting arise from a misalignment between institutional practices and learner diversity rather than from individual deficits alone. The recurring descriptions of rigid assessment methods, limited access to assistive technologies, and uneven faculty awareness indicate that students’ capabilities to achieve valued educational outcomes are constrained not only by their impairments but also by the ways in which teaching, assessment, and support systems are organized. From a capability approach viewpoint, these conditions restrict students’ opportunities to demonstrate their knowledge, to participate fully in class, and to exercise agency over their learning, even when they are motivated and academically able. By conceptualizing the results as structured variation in how students and faculty understand support for LDs—ranging from remedial, individually targeted help to more systemic, rights-based arrangements—the study aligns the phenomenographic outcome space with broader international debates about how higher education institutions can move from access to genuinely inclusive participation.

From the results of the current study and from previous studies, there are several approaches that can be adopted to address challenges faced by students with LDs. For instance, Svendby (2020) suggests that, on the one hand, the issue can be approached from a perspective according to which professional performance is humanized, and students with LDs can be approached with empathy, while on the other hand, a student’s disability can be seen as an opportunity to work more effectively with people who have varying abilities. The social and educational programs designed to address challenges faced by students with LDs support inclusive education and affect the way teachers instruct and the strategies they adopt to improve teaching and learning processes, spreading an ethos of support for the student (Sulaj et al., 2021).

In Saudi Arabia, a commitment to humanism and equal rights occupies a prominent place and is reflected by an ongoing focus of the government on ensuring that everyone in the country lives a decent life. In this regard, those with LDs are considered an important group in society and, therefore, have their needs prioritized by the Ministry of Education, in principle if not always in practice. To this end, the Ministry of Education is implementing programs to make educational services more accessible to students with LDs. The Ministry of Education is also responsible for setting standards for evaluating the preparedness of teachers to accommodate students with LDs (Education and Training Evaluation Commission, 2023). By developing formal guidelines for teachers and individuals with LDs in Saudi Arabia, the Ministry of Education engages in efforts to develop the expertise and skills of students with LDs. This commitment is shown by the ongoing training courses in modern teaching methods. Added to this, higher education institutions engage in efforts to ensure that they hire faculty specialized in LDs. If there is success in this area, it can be posited that the country’s education system will have managed to advance the objectives of Saudi Vision 2020, which seeks to provide an appropriate learning environment for all students, including students with LDs.

## 5. Limitations and Conclusions

As with all educational research, the present study has limitations. For example, the sample size is small, which means that its results may not be generalizable to a larger population of university students with LDs. Moreover, the study is limited to students and faculty at a single university, with the result that the experiences captured in this study may not be representative of students with LDs in all Saudi institutions. In addition, due to ethical constraints, we could not collect information that identified the specific LD of a given participant. Neither could we collect information about the specific LD with which each faculty member was familiar. Instead, we relied on general reference to LD, and this represents a limitation of the current research. We encourage future research on specific LDs, on the presumption that each type of LD may present unique challenges alongside challenges that may accompany most LDs.

The present study identified challenges to participation and success for students with LDs in higher education in Saudi Arabia. From the literature reviewed and the views expressed by the current participants, it can be concluded that students with LDs need academic support, collaboration, and understanding from different role players in the university, including faculty members and peers. It was also noted that high schools can play a significant role in ensuring that when students with LDs arrive at universities, they are adequately prepared. The present study’s results also highlight the importance of ensuring that the university community is aware of the needs and challenges faced by students with LDs. For instance, the awareness that could be inspired by insights generated by the present study could lead to the development of policies and programs that better support students with LDs. Such policies and the support they provide to students with LDs could promote a more inclusive environment in which equality in educational opportunities is possible, including for students with LDs. To address the challenges faced by students with LDs, there is a need to go beyond making academic support services available to also providing emotional and psychological support to assist students in achieving their potential. It is, therefore, important for universities to develop and implement strategies for encouraging students with LDs to disclose their disabilities as soon as possible.

### Recommendations

Based on the results of the present study, several recommendations can be made. For instance, because many of the identified challenges emanate from the lack of awareness among different role players in the university setting, training that includes increasing awareness is important. This will assist stakeholders in becoming more aware of the support required by students with LDs. For policymakers, awareness training could result in the development of policies that support diversity at universities. Although scholars such as Hopkins (2011) suggest that this training should be mandatory and ongoing, there is a need for research on how this training can be implemented in practice.

The following are additional recommendations, based on the insights obtained from this study:

- Revise teaching strategies, and study and assessment methods, to ensure equitable treatment of students with LDs, for example, by providing special software and extra time to complete tasks.
- Design tailored academic support programs, including individual motivation sessions and tutoring, to assist students with LDs in developing skills that will lead to better academic performance.
- Encourage students with LDs to participate in extracurricular university activities as a way of promoting effective communication, developing social skills, and increasing a sense of belonging.

It is anticipated that implementing these strategies can help universities establish an inclusive learning environment that will facilitate all students, including those with LDs, to improve their academic achievement.

## Figures and Tables

**Table 1:** Available participant demographics (*n* = 9).

Participant ID	Age	Sample Classification	Teaching Experience (Years)
Participant (1)	20	Student with LD	NA
Participant (2)	22	Student with LD	NA
Participant (3)	23	Student with LD	NA
Participant (4)	24	Student with LD	NA
Participant (5)	25	Student with LD	NA
Participant (6)	35	Faculty member	5
Participant (7)	45	Faculty member	11
Participant (8)	49	Faculty member	15
Participant (9)	55	Faculty member	20

*Notes*: LD = Learning disability; NA = Not applicable.

## Data Availability

The data presented in this study are available on request from the corresponding author due to ethical constraints.
